# Negation interacts with motivational direction in understanding action sentences

**DOI:** 10.1371/journal.pone.0234304

**Published:** 2020-06-22

**Authors:** Hipólito Marrero, Sara Nila Yagual, Elena Gámez, Mabel Urrutia, Jose Miguel Díaz, David Beltrán

**Affiliations:** 1 Departamento de Psicología Cognitiva, Social y organizacional, Universidad de La Laguna, La Laguna, Spain; 2 Instituto de Neurociencias de la Universidad de La Laguna, La Laguna, Spain; 3 Facultad de Ciencias Sociales y de la Salud, Universidad Estatal Península de Santa Elena, La Libertad, Ecuador; 4 Facultad de Educación, Universidad de Concepción, Concepción, Chile; Newcastle University Institute for Health and Society, UNITED KINGDOM

## Abstract

Linguistic negation acts by inhibiting the representation of information under its scope, often leading to the representation of positive alternative states of affairs. Motivational direction refers to approach/avoidance intentionality in our interactions with environmental stimuli expressed by means of verbs (e.g., “accept” vs “reject”). We consider it plausible that negation interacts with direction to represent the true motivation of the protagonist in sentence understanding (e.g., if an approach action is negated it is represented as avoidance). In the first study, we examine this interaction offline by asking participants to judge approach or avoidance meaning of affirmative (e.g., “he/she included/excluded meat”) and negative sentences (“he/she did not include/exclude meat”). Results support that negation reversed participants’ interpretation of sentence motivational direction. In a further study, we carried out two probe recognition experiments to examine the interaction during sentence comprehension; in both, the critical probe was the word referring to the target of the action (e.g., “meat”). In the first experiment, participants had to recognize the probe word presented 1500 milliseconds after sentence offset, while for the second one, the delay was 500 milliseconds. Results showed that at 1500 ms, target recognition took significantly more time for negated avoidance sentences than for the other conditions. Therefore, representing negated avoidance sentences seems to imply more complex processing, as avoidance verbs would be implicitly negative. By contrast, at the 500 ms delay, negation impaired target recognition for both approach and avoidance sentences, suggesting an unspecific inhibitory effect of negation at that sentence processing stage. Implication of these results for both research on negation and in action understanding are discussed.

## Introduction

Negation is an important linguistic operator (see [1]). Previous research has examined its role in the comprehension of verbal utterances and sentences (see [2]). From a cognitive point of view, negation could affect information processing in different ways. On the one hand, negation induces a partial inhibition of the representation of the negated concept (for example, to indicate no execution in the case of overt motor actions as in “Petra did not clean the dishes”) (see [3]). On the other hand, negation could be interpreted in terms of its implications; for example, when negated information pertains to a binary category: “if the number is not even”, the implication is spontaneously made that the number is odd (see [4, 5, 6]). These effects are consistent with the prevailing cognitive model on negation processing, the so-called two-step model [7, 5, 8]. This model, which assumes the more general Embodied Simulation Theory (EST, [9, 10, 11], decomposes the comprehension of negative sentences into two sequential steps, distinguishing them by the content of the involved representations: the negated situation (e.g., a ‘closed door’ for the sentence *The door is not closed*) for the first step, and a second step where this state of affairs is rejected. In this step, implications about alternatives to the rejected state of affairs (e.g., an ‘open door’) could be triggered and the alternative be represented by simulation [12, 13, 14, 8, 15]. In accordance with the model, this is predicted to occur when negation affects a binary category or the predicates are contradictory, which enables the availability of alternatives; for example: an “open door” for “the door is not closed” (see [8]).

One relevant characteristic of language is that it provides a reference for individuals’ attitudes either in favor or against environmental stimuli by means of verbs like accept vs. reject, praise vs. despise, approve vs. criticize, support vs. censor, or care vs. abandon. The stimuli could be either other people, as in this example: “John included/excluded Ruth in/from his group of friends”, or things: “John included/excluded meat in/from his shopping list”. These verbs are relatively abstract in that they describe intentionality in our interactions with environment targets, rather than specific action patterns. For example, the utterance “she excluded meat in her eating” describes avoidance intentionality towards meat, but not the specific action, which might be instantiated in variety of ways: i.e., asking for vegetarian dishes in restaurants, or shopping at vegetarian stands in the market. Approach is mostly associated with actions pro stimulus evaluated as positive (e.g., beneficial for good health)–to keep it close (literally or figuratively). Whereas, stimuli evaluated as negative are generally avoided, and hence associated with actions against stimulus–to push it away [16]. Thus, approach and avoidance intentions constitute a semantic frame or category to be encoded for action understanding [17, 18].

In this paper, we examine for the first time the role of negation in comprehension of verbal actions of approach and avoidance. In particular, we analyze how motivational direction, as conveyed by approach and avoidance verbs, might interact with linguistic negation, placing emphasis on the representational and processing consequences of this interaction, which lays the foundations for the empirical work that will be described after that.

## Interaction between negation and direction in action-sentence understanding

First of all, at the representational level, we consider that approach and avoidance concepts present some commonality with the distinction between affirmation and negation. Approach verbal actions resemble affirmation, whereas avoidance would be represented as implicitly negative. As mentioned, representation of avoidance verbs would involve an agent’s attitude against the approach of the stimulus towards themselves. In support of this idea, negation, both bodily (head shaking) and linguistically expressed, develops early to communicate an attitude of rejection ("protest") towards disliked stimuli (see [19]; see also [20, 21]). Thus, plausibly by overlearning, verbal representations of avoidance actions could involve that avoidance is represented “against stimulus approach”; given that “against” is negative [19, 22], avoidance verbs would be implicitly represented as “no-approach”.

Secondly, we consider the interaction between motivational direction and negation at the level of processing in action-sentence comprehension. In accordance with the two-step model, availability of sentence information goes through a process of negation integration in the meaning of the sentence (see [2]). Early on during comprehension (i.e., during the first step), the negated situation is being simulated (“meat included” in the example “John did not include meat in his shopping list”) and then the target term “meat” is highly available as in the affirmative version of the sentence. Then, when it comes to the second step, the simulation of the negated situation gets inhibited and accordingly the target term is low in availability. Finally, the comprehender releases the inhibition, and simulates the actual situation (“meat excluded”), and thus availability of the target term is back to base line.

The effects of inhibition and representation of the alternative situation have been made evident in the context of a probe recognition task paradigm. In this task, participants must decide whether a probe (a word or a picture) has been shown in the preceding sentence; it is a task of immediate memory recognition. The key measure is the latency for making the decision, which is taken as an index of the activation level for the concept associated with the probe (see [2]). The delay between sentence display and the recognition probe plays an important role in the effect of negation [8, 5, 2]. As we are interested in the more direct effect of negation on understanding motivational sentences, we focus this study on step 2, and so we target the middle and late effect. A longer delay of 1500 ms has been shown to be sufficient for integration of negation in the sentence meaning. Thus, it facilitates the effect of negation in triggering the representation of positive alternatives for comprehension when they are available. By contrast, at intermediate delays (around 500–1000 ms), sentential negation has an inhibitory effect and slows recognition of information under its scope.

The effect of negation on triggering the representation of the positive alternative highlights a feature of motivational direction that we have not directly addressed yet, and which is crucial to understand how it might interact with negation. Motivational direction is a binary conceptual category, that is, a category composed of two opposing meanings. When we state our avoidance intentionality towards something (for example “excluding meat in in your diet”) this implies that we do not approach it (by including it in the example), and vice-versa in the case of approach intentionality; if we approach meat (“including meat in your diet”) this implies that we do not avoid it (by excluding it). Certainly, at the level of our reaction towards stimuli we could feel both approach and avoidance tendencies [23]. For example, it could be that “eating meat” continues to be appealing to us in spite of our intention of avoiding it. Yet, considering the intentionality of the action, this can be either approach or avoidance, but likely not both at the same time. Thus, negating an approach action (“did not include”) would trigger the implication of the alternative, namely an avoidance action and then lead to the representation of a suitable contextual action (e.g., “excluded”); and vice versa for the negation of avoidance (“did not exclude”) would trigger the implication of the alternative motivation and then lead to the representation of a suitable contextual action (e.g., “included”).

This process would be modulated by approach and avoidance content of sentences. Previous research has supported that sentences that contain negatives are more difficult to process than affirmative ones [24, 25, 4, 8, 26, 27] and sentences become increasingly difficult to process the more (explicit or implicit) negations they contain [28, 29, 17, 30]. As mentioned, representation of avoidance as “no-approach” might involve an implicit negation. Thus, negated avoidance sentences involve more cognitive complexity for comprehension related to processing something like a double negation. Greater complexity would make it more difficult to integrate sentential negation in the sentence meaning, and, in turn, build up a representation of the actual situation. Thus, inhibition is still in effect, and the time taken for probe recognition would be longer after negative avoidance sentences than after negative approach sentences even with a long recognition delay.

Here, we present a study aimed at examining the interaction between negation and motivational direction in sentence comprehension. For this purpose, we reused approach and avoidance sentences of a prior study ([18], see also [31, 32]), and introduced a manipulation of the polarity of the sentence. Firstly, we carried out a study where participants had to judge offline approach or avoidance meaning of sentences both in the affirmative and the negative version (see examples in Table 1). This study serves to examine if participants’ interpretations of negative sentences are consistent with the predicted interaction between polarity and motivational direction. However, the main purpose of our research is to evaluate this interaction for sentence comprehension in the context of a probe recognition task. In Experiment 1, participants were presented with approach and avoidance sentences, either affirmative or negative. The probe to recognize was the action target (e.g., “meat”), and the delay between sentence display and the recognition probe was 1500 ms. In Experiment 2, we repeated the same design with a intermediate delay of 500 ms. Our main hypotheses were as follows:

**Table 1:** List of approach and avoidance sentences (verb + target) in affirmative and negative version with approximate translation into English.

AFFIRMATIVE		NEGATIVE	
APPROACH	AVOIDANCE	APPROACH	AVOIDANCE
Incluyó el pan (He/she included bread)	Excluyó el pan (He/she excluded bread)	No incluyó el pan (He/she did not include bread)	No excluyó el pan (He/she did not exclude bread)
Se quedó con el coche He/she kept the car	Se desprendió del coche (He/she got rid of the car)	No se quedó con el coche (He/she did not keep the car)	No se desprendió del coche (He/she did not get rid of the car)
Eligió la carne (He/she chose meat)	Rechazó la carne (He/she rejected meat)	No eligió la carne (He/she did not choose meat)	No rechazó la carne (He/she did not reject meat)
Aceptó el recibo (He/she accepted the receipt)	Descartó el recibo (He/she discarded the receipt)	No aceptó el recibo (He/she did not accept the receipt)	No descartó el recibo (He/she did not discard the receipt)
Aceptó la sugerencia (He/she accepted the advice)	Rechazó la sugerencia (He/she rejected the advice)	No aceptó la sugerencia (He/she did not accept the advice)	No rechazó la sugerencia (He/she did not reject the advice)
Apoyó el logo (He/she supported the logo)	Se opuso al logo (He/she opposed the logo)	No apoyó el logo (He/she did not support the logo)	No se opuso al logo (He/she did not oppose the logo)
Aprobó el estilo (He/she approved the style)	Criticó el estilo (He/she criticize the style)	No aprobó el estilo (He/she did not approve the style)	No criticó el estilo (He/she did not criticize the style)
Cuidó el jardín (He/she cared for the garden)	Abandonó el jardín (He/she neglected the garden)	No cuidó el jardín (He/she did not care for the garden)	No abandonó el jardín (He/she did not neglect the garden)
Mantuvo la hipótesis (He/she kept the hypothesis)	Eliminó la hipótesis (He/she got rid of the hypothesis)	No mantuvo la hipótesis (He/she did not keep the hypothesis)	No eliminó la hipótesis (He/she did not get rid of the hypothesis)
Guardó el álbum (He/she saved the album)	Tiró el álbum (He/she threw out the album)	No guardó el álbum (He/she did not save the album)	No tiró el álbum (He/she did not throw out the album)
Aprobó el proyecto (He/she supported the project)	Censuró el proyecto (He/she censored the project)	No aprobó el proyecto (He/she did not support the project)	No censuró el proyecto (He/she did not censor the project)
Disfrutó con el juego He/she enjoyed the game)	Se disgustó con el juego (He/she got upset with the game)	No disfrutó con el juego He/she did not enjoy the game)	No se disgustó con el juego (He/she did not get upset with the game)
Apreció la música (He/she praised the music)	Despreció la música (He/she despised the music)	No apreció la música (He/she did not praise the music)	No despreció la música (He/she did not despise the music)
Mantuvo la lectura (He/she kept reading)	Quitó la lectura (He/she removed reading)	No mantuvo la lectura (He/she did not keep reading)	No quitó la lectura (He/she did not remove reading)

H1. With the long delay of 1500 ms, recognition times will be significantly longer in avoidance negative sentences than in approach negative sentences. Although this long delay is assumed to enable the integration of negation in the meaning of the sentences, negative avoidance sentences are more complex because of the implicit negation brought about by avoidance verbs. Greater complexity would hamper building up a representation of the actual situation, and inhibition is still in effect. Therefore, the time taken for probe recognition would be longer in negative avoidance sentences than in negative approach sentences.

H2: With the intermediate delay of 500 ms, recognition times will be significantly longer in negative sentences than in affirmative sentences. This reflects the process of a general inhibition of negation on the representation of a negated situation, assumed to occur at the beginning of the second step of the two-step model.

## Study 1: Evaluation of direction of action-sentences

The aim of this study was to test whether action sentences (verb + target) were interpreted as approach or avoidance, and more specifically whether sentences with negated approach verbal predicates were judged as avoidance direction, whereas negated avoidance verbal predicates were judged as approach direction.

### Methods

#### Stimuli

We selected a pool of approach and avoidance sentences from Marrero et al. ([18]; see also [32, 31]); verbs were in past tense and third person singular. Approach and avoidance action verbs were varied, for example, reject, exclude, choose, accept, include, …. Approach and avoidance verbs were semantically related in each sentence version (i.e. “accepted vs. rejected”). To make the negative version, we added an explicit negation to the verbal predicate in the affirmative approach and avoidance versions. Approach and avoidance verbs could appear with different targets (between 3 and 5). In Table 1, the list of approach vs. avoidance verbs with one of the targets is shown.

#### Participants and procedure

Thirty psychology students at the University of La Laguna (18 females, mean age: 20.4 years old) participated in this study in exchange for course credits. They were told to evaluate the direction (approach or avoidance) of pairs of sentences, either affirmative or negative, with the same target: “your task is to point out in each pair which sentence has an approach sense, and which has an avoidance sense”. The sentences refer to actions performed by a third person. For example, in this pair “he/she included bread vs. he/she excluded bread”, or in the negative pair version “he/she did not include bread vs. he/she did not exclude bread” to choose which sentence means approach and which avoidance. In the instructions, approach was described as the action of having the sense of approaching the thing towards oneself, or the approach of oneself towards the thing, either physically or affectively, and involved a positive attitude. Avoidance was described as the action of having the sense of keeping a thing away from oneself, or keeping oneself away from the thing, either physically or affectively, and involved a negative attitude (see [16]).

### Results and discussion

We found that approach/avoidance direction was correctly identified, 98.91% of times (SD = 2.33), in pairs of sentences in affirmative versions. In the case of negative versions, sentences with negated approach verbs were judged as involving avoidance direction, whereas sentences with negated avoidance verbs were judged as involving approach direction in 98.20% of the times (SD = 3.78). T-test comparisons showed that there was no significant difference between these percentages, *p* > 0.10. These results suggest that when evaluated offline, negated approach sentences were judged as avoidance and negated avoidance sentences were judged as approach.

## Experiment 1: Probe recognition with 1500 ms delay

A usual paradigm to examine integration of negation in sentence meaning is recognition latency for previously read negated information (see [8]). In this experiment, we tested the effect of negation on immediate recognition of the action target. The delay between the end of sentence display and the appearance of the action target for recognition was 1500 ms.

### Methods

#### Participants

Eighty-nine healthy students of Psychology at the University of La Laguna participated in this experiment (70 females, mean age: 20.1 years-old) in exchange for course credits. All participants were right-handed, and none of them participated in the Study 1. This experiment was carried out in accordance with the recommendations of the Committee of Ethics of Research and of Animal Welfare of University of La Laguna and was approved by this Committee. Participants agreed with their participation and signed an informed consent form.

#### Stimuli

Experimental sentences were developed from sentences of Study 1, in which a person noun as the action subject and some contextual information after the target were added (see Table 2).

**Table 2:** List of approach and avoidance sentences in affirmative version with approximate translation into English, and examples of filler sentences.

APPROACH	AVOIDANCE	TARGET (Recognition)
Julio **incluyó el pan** en la lista de la compra (Julio included bread in the shopping list)	Julio **excluyó el pan** de la lista de la compra (Julio excluded bread in the shopping list)	PAN
Teresa **se quedó con el coche** por su aspecto exterior Teresa kept the car for its exterior appearance	Teresa **se desprendió del coche** por su aspecto exterior (Teresa got rid of the car for its exterior appearance)	COCHE
Gabriel **eligió la carne** en el menú del restaurante (Gabriel chose meat in the restaurant menú)	Gabriel **rechazó la carne** en el menú del restaurante (Gabriel rejected meat in the restaurant menú)	CARNE
Petra **aceptó el recibo** del banco de la localidad (Petra accepted the receipt of the bank of the town)	Petra **descartó el recibo** del banco de la localidad (Petra discarded the receipt of the bank of the tow	RECIBO
Daniel **aceptó la sugerencia** en el restaurante del centro (Daniel accepted the advice in the restaurant of the center)	Daniel **rechazó la sugerencia** en el restaurante del centro (Daniel rejected the advice in the restaurant of the center)	SUGERENCIA
Cristian **apoyó el logo** del club de deporte (Cristian supported the logo of the sport club)	Cristian **se opuso al logo** del club de deporte (Cristian opposed the logo of the sport club)	LOGO
Ricardo **aprobó el estilo** de la casa del barrio (Ricardo approved the style of the neighborhood house)	Ricardo **criticó el estilo** de la casa del barrio (Ricardo criticize the style of the neighborhood house)	ESTILO
Sofía **cuidó el jardín** de su casa de campo (Sofía cared for the garden of the countryside house)	Sofía **abandonó el jardín** de su casa de campo (Sofía neglected the garden of the countryside house)	JARDÍN
Irene **mantuvo la hipótesis** en el experimento de ciencias (Irene kept the hypothesis in the science experiment)	Irene **eliminó la hipótesis** en el experimento de ciencias (Irene got rid of the hypothesis in the science experiment)	HIPÓTESIS
Mónica **guardó el álbum** de la familia con intención (Mónica saved the family album with intention)	Mónica **tiró el álbum** de la familia con intención (Mónica threw out the family album with intention)	ALBUM
Pablo **aprobó el proyecto** de innovación del Estudio (Pablo supported the innovative project of the Studio)	Pablo **censuró el proyecto** de innovación del Estudio (Pablo censored the innovative project of the Studio)	PROYECTO
Alejandro **disfrutó con el juego** en el partido de fútbol (Alejandro enjoyed the game in the football match)	Alejandro **se disgustó con el juego** en el partido de fútbol (Alejandro got upset with the game in the football match)	JUEGO
Juan **apreció la música** de la banda de la ciudad (Juan praised the music of band of the town)	Juan **despreció la música** de la banda de la ciudad (Juan despised the music of band of the town)	MÚSICA
Rebeca **mantuvo la lectura** entre sus actividades de ocio (Rebeca kept reading among her leisure activities)	Rebeca **quitó la lectura** de sus actividades de ocio (Rebeca removed reading among her leisure activities)	LECTURA
**FILLER (examples)**		
Elvira potenció el volumen de la radio en su habitación (Elvira boosted the volume of the radio in her room)		COMEDOR
Lorena sufrió el bullicio de la avenida con nerviosismo (Lorena suffered the bustle of the avenue with nervousness)		TRÁFICO

Fifty-one participants (36 females, mean age: 21.3 years-old), none of them involved in either the Experiment or in Study 1, evaluated the imaginability of approach and avoidance sentences on a rating scale from 1 (“abstract”) to 5 (“concrete”). Results are shown in Table 3. We carried out an ANOVA on sentences’ imaginability ratings taking Polarity (affirmative vs. negative) and Direction (approach vs. avoidance) as within-subject factors. The main effect of Polarity attained significance, *F* (1, 50) = 12.05, *p* = 0.001, *η*^*2*^ = .194. Affirmative sentences (*M* = 3.01, SD = 0.48) were rated as slightly more imaginable than negated sentences (*M* = 2.78, SD = 0.49). No main effect of Direction was found, *p* > .20. The polarity x direction interaction was also significant, *F* (1, 50) = 9.54, *p* = .003, *η*^*2*^ = .160. Planned comparisons showed that negated avoidance sentences were evaluated as slightly less imaginable than the other statements: negated approach sentences (*Mean Diff* = -0.18, SD = 0.53, *t*(50) = -2.50, *p* = .016); avoidance sentences (*Mean Diff* = -0.38, SD = 0.69, *t*(50) = -3.98, *p* < .0001); and approach sentences (*Mean Diff* = -0.26, SD = 0.66, *t*(50) = -2.85, *p* = .007).

**Table 3:** Means and standard deviations (in parenthesis) of recognition task latencies for correct responses as a function of Direction and Polarity (delay of 1500 ms). Imaginability scores of sentences are placed in bold under each condition.

Polarity			
Direction	Affirmative (A)	Negative (N)	A-N
Approach (Ap)	698 (134) **2.95 (.54)**	689 (108) **2.87 (.52)**	09
Avoidance (Av)	702 (119) **3.08 (.53)**	720 (136) **2.69 (.58)**	-18
Ap-Av	-4	-31	

As can be seen, sentences in the different versions were evaluated close to “neither concrete nor abstract (point 3 on the scale)”, affirmative sentences were rated as slightly more imaginable than negated sentences, and negated avoidance sentences showed slightly less imaginability than the other versions. Length and syllabic length were checked for the approach and avoidance sentences. Pairwise comparison showed no significant differences between them, p > .05.

#### Design and procedure

A 2 x 2 within-subject factorial design was used, with Direction and Polarity of sentence as factors. Participants were told to read sentences that appeared while seated in front of a computer screen. Each sentence presentation started with a cross point displayed in the middle of the screen for 750 ms. Following an interval of 150 ms, one sentence was displayed. Sentence presentation was segmented as in the following example: “*Petra/aceptó/el/recibo/del/banco/de la/localidad*” (“Petra/accepted/the/receipt /of the/bank/of the/town”); in the negative version: “*Petra/no aceptó/el/recibo/del/ banco/de la/localidad*” (“Petra/did not accept/the/receipt/of the/bank/of the/town”). Each segment was displayed during 300 ms with an interval of 150 ms between them. After the sentence was displayed, participants were presented with a word and told to respond whether or not the word was in the sentence. This word appeared 1500 ms after the sentence display ended. Participants were told to respond either affirmatively or negatively by pressing key P and key Q on the keyboard, respectively. The word remained on the screen for 3000 ms or until a response was made. The interval between each sentence display was 750 ms. Participants were given 140 sentences, 20 for each experimental condition and 60 filler sentences. Filler sentences were thematically similar to experimental sentences with affirmative and negative versions. In this way, participants read a greater variety of verbal actions and contexts.

In the experimental sentences, the word to be recognized was the target, and the correct response was always affirmative, whereas in the filler sentences the correct response was negative (the word did not appear in the sentence). To avoid participants focusing exclusively on the superficial detection of the verb complement noun, one quarter of the sentences (36) were immediately followed by a question on the content just read (e.g., “Is it stated that Petra rejected the receipt of the bank?”). This question had either a positive or a negative response half of the time and remained on the screen for 5000 ms or until a response was made. Feedback was given to the participants and displayed for 2000 ms. After a delay of 750 ms, a new sentence was displayed.

Participants were randomly assigned to one of the sets of sentences resulting from the counterbalance of the experimental conditions. This ensured that every participant received an equal number of sentences for each of the four conditions, and no participant received the same sentence twice. Sentences were randomly presented to the participants in each of the counterbalancing sets.

### Results and discussion

We carried out an ANOVA on recognition latencies for correct responses with Direction and Polarity as within-subject factors. Recognition latencies above/under 2.5 SD of the participant mean (4.9%) were removed from the analysis. Moreover, participants’ mean latencies over or under 2 SD of the group mean in each condition were substituted for the group mean in the condition (1.7%). Two participants were removed due to their average mean latencies being over 2 SD of the group mean over the conditions. Recognition latencies in the different conditions are shown in Table 3.

The effect of Direction was significant, *F* (1, 86) = 9.68, *p* = .003, *η*^*2*^ = .101. Recognition latencies were shorter in approach sentences (*M* = 694, SD = 115) than in avoidance sentences (*M* = 711, SD = 121). No main effect of Polarity was found. The direction x polarity interaction was significant, *F* (1, 86) = 5.41, *p* = .022, *η*^*2*^ = .059. Planned comparisons showed that negative avoidance sentences took more time for target recognition than the other conditions: with approach sentences *(Mean Diff* = 21.76, SD = 71.35, *t*(86) = 2.84, *p* < .006); with negative approach sentences (*Mean Diff* = 31.25, SD = 78.11, *t*(86) = 3.73, *p* < .000); and with avoidance sentences (*Mean Diff* = 18.28, SD = 81.59, *t*(86) = 2.09, *p* < .040). No other comparisons attained significance.

As can be seen, the main effect of Direction was qualified by the direction x polarity interaction showing that negation significantly produced greater latencies in avoidance than in approach sentences. This interaction suggests that integrating negation in the meaning of the sentence is easier for approach than for avoidance.

Overall, the percentages of correct responses were high (93%). We carried out an ANOVA on percentages of correct responses in the recognition task with Direction and Polarity as within-subject factors. Neither the main effects of Direction and Polarity nor the direction x polarity interaction were significant, *p* > .10.

## Experiment 2: Probe recognition with 500 ms delay

According to Kaup et al. [8], in the representation of the alternative situation, negation induces a rejection of the negated situation as the true situation. Thus, negation would impair target recognition in both approach and avoidance sentences compared to the affirmative version at a certain moment after sentence reading before the delay of 1500 ms. This is a relevant prediction to support an EST interpretation of our results. With the aim of testing this prediction, we carried out an experiment where the delay between reading and the display of the recognition probe was shorter: 500 ms.

### Methods

#### Participants

Forty healthy students of Psychology at the University of La Laguna participated in this experiment (32 females, mean age: 18.71 years-old) in exchange for course credits. All participants were right-handed, and none of them had participated in Study 1 nor in Experiment 1.The sample size was calculated as the minimum for a small-expected effect [33]. This experiment was carried out in accordance with the recommendations of the Committee of Ethics of Research and of Animal Welfare of University of La Laguna and was approved by this Committee. Participants agreed to participate and signed an informed consent form.

#### Stimuli

As in Experiment 1.

#### Design and procedure

As in Experiment 1. The probe word appeared 500 ms after the sentence display ended.

### Results and discussion

We carried out an ANOVA on recognition latencies for correct responses with Direction and Polarity as within-subject factors. Recognition latencies above/under 2.5 SD of the participant mean (7.5%) were removed from the analysis. Moreover, participants’ mean latencies over or under 2 SD of the group mean in each condition were substituted for the group mean of the condition (2.5%). One participant was removed due to their average mean latencies being over 2 SD of the group mean in all the conditions. Recognition latencies in the different conditions are shown in Table 4.

**Table 4:** Means and standard deviations (in parenthesis) of recognition task latencies for correct responses as a function of Direction and Polarity (delay of 500 ms).

Polarity			
Direction	Affirmative (A)	Negative (N)	A-N
Approach (Ap)	656 (75)	678 (83)	-22
Avoidance (Av)	672 (78)	679 (78)	-7
Ap-Av	-16	-1	

The effect of Polarity was significant, *F* (1, 38) = 4.79, *p* = .035, *η*^*2*^ = .112. Recognition latencies were shorter in affirmative sentences (*M* = 664, SD = 71) than in negative sentences (*M* = 679, SD = 75). No significant effect was found either for Direction or for the interaction direction x polarity, *p* > .20.

We also carried out an ANOVA on recognition latencies for correct responses with Direction and Polarity as within-subject factors, and the delay (1500 vs 500 ms) as between-subjects factor. The effect of Direction was significant, *F* (1, 124) = 7.28, *p* = .008, *η*^*2*^ = .055. Recognition latencies were shorter in approach sentences (*M* = 685, SD = 104) than in avoidance sentences (*M* = 700, SD = 109). Likewise, there was a significant effect of the direction x polarity x delay interaction, *F* (1, 124) = 4.94, *p* = .028, *η*^*2*^ = .038. This interaction mainly showed larger recognition latencies for negative-approach (*M* = 678, *SD* = 83) than for affirmative-approach (*M* = 656, *SD* = 75) sentences in the delay of 500 ms, *t*(38) = - 2.42, *p* = .020. By contrast, in the delay of 1500 ms, negative-avoidance sentences took more time for target recognition than the other conditions, as described in Experiment 1.

## General discussion

In this paper, we have examined the interaction between polarity (affirmative vs. negative) and direction (approach vs. avoidance) in understanding action-sentences. Study 1 showed that, when evaluated offline, approach and avoidance were correctly identified regardless of the polarity of the sentences, either affirmative or negative. This suggests that, for negative sentences, approach verbs were judged as avoidance whereas the avoidance ones were judged as approach. This result is in accordance with the expected interaction between negation and direction, where negation would trigger the implication of the alternative motivation to the negated one. However, this evidence is weak inasmuch as our task forced the approach/avoidance judgment, without having the opportunity of selecting a neutral option: “neither approach nor avoidance”. Instead of implicating an alternative motivation, negation could have an effect of weakening approach or avoidance evaluation towards more neutral values (see [34]).

More importantly, we examine negation and motivational direction interaction during comprehension by testing its effect on immediate action target recognition in two experiments. Two different effects were expected. On the one hand, negation would lead to the representation of the positive motivational alternative but the effect of negation on the representation of the motivational alternative could be different in avoidance versus approach sentences, since avoidance verbs are implicitly negative. Thus, impairment of recognition latencies was expected for negated avoidance sentences in contrast to negated approach ones, because of a double negative-like effect. On the other hand, sentential negation would lead to the inhibition of the representation of negated actions, and so greater latencies are expected for target recognition in negative versus affirmative sentences. The occurrence of one or the other effect would depend on the delay between sentence display and the recognition probe [35; 8; 36].

In accordance with Hypothesis 1, at a delay of 1500 ms (Experiment 1), we found that target recognition took significantly more time for negated avoidance than for negated approach sentences. By contrast, with a shorter delay of 500 ms (Experiment 2), and consistent with Hypothesis 2, negation impaired target recognition of both approach and avoidance sentences. The contrast between experiments 1 and 2 suggests that at a delay of 500 ms negation causes inhibition of the representation of negated actions, both of approach and avoidance. According to the two-step model, this could be the result of rejection of the negated action as the true action at the beginning of the second step [8]. At the end of the second step, implications about alternatives to the rejected state of affairs could be elicited [12, 13, 14, 8; 15]. This is predicted to occur when negation affects a binary category, as is the case in approach and avoidance motivational sentences. In our motivational directional sentences, following the implication that a “no-approach action” implies an “avoidance action”, and vice-versa a “no-avoidance action” implies an “approach action” a positive representation of the alternative situation (e.g., “excluded” in the case of “did not include”) would be built up. At 1500 ms delay, negation would have already been integrated into sentence meaning, and plausibly a positive alternative action would be represented in approach sentences, but this process would not have happened in avoidance sentences at this time.

For approach sentences, which are relatively simple, these two processes are reflected in the intermediate and the long delay respectively. In the intermediate delay condition, negation leads to longer response times in the probe-recognition task because of inhibition, whereas in the long delay condition, inhibition is already abolished because the reader has successfully integrated the negation and now focuses on alternative situations and response times are therefore not prolonged. Avoidance sentences in contrast are assumed to be much more complex, as they are implicitly negative resulting in a double negation in the negative conditions. Building up the meaning of negated avoidance sentences implies more complex processing, takes more time and thus negation (and its inhibitory effect) is retained for longer in sentence representation, which prolongs response times even in the long delay condition.

More complex processing could be also related to the possibility that negation of avoidance does not convey representations that are equivalent to the corresponding affirmative meaning (approach). In accordance with fixed or flexible theories about simulation, negation of lexical (literal) elements can be included alongside the simulation, and the marker (negation) is somehow retained in the representation (e.g., Mental models: [37, 6]). Likewise, sometimes it is preferable to use more propositional representations (less based on experience) to build up the meaning of sentences. In contrast to negated approach sentences, negated-avoidance sentences would be represented with negation being retained in the representation of the sentence. As a consequence, inhibition would be exerted on the avoidance action representation, which would slow target recognition latencies.

We have found here that negation of avoidance sentences produces longer probe recognition latencies than negation of approach at a delay of 1500 ms, and longer recognition latencies for both approach and avoidance sentences at a delay of 500 ms. Interestingly, it is consistent with the imaginability ratings reported by a different sample of participants for our sentences, i.e., with how easily they could mentally imagine the described situation: negative sentences and negated avoidance sentences are more difficult to imagine. Imaginability can be interpreted in this context as a broad-brush index of the extent to which participants are able to ground sentence meaning on concrete experiences. The higher the imaginability of the sentence, the easier it should be to build an embodied simulation of the sentence. Together, imaginability ratings and recognition latencies suggest that negation makes it harder to build up the representation of the situation being described. This is in accordance with the two-step model of negation processing [5]. In addition, the representation of an alternative situation is more difficult in avoidance sentences as they are implicitly negative and involve handling a double negation.

## Limitations and future research

One limitation of our research is the fact that our immediate recognition task provides easy accessibility of the probe item inasmuch as it has been explicitly mentioned in the sentence. This could explain the small size of the effects found. Other versions of the task where recognition is more demanding (for example probe-word naming) could be used that increases the size of the negation effect on target recognition. Likewise, other delays (see [8]) should be tested in order to examine whether or not negation triggers the representation of the alternative motivation during comprehension of avoidance sentences, or if this were not to happen at least it was explicitly required. Moreover, our offline approach/avoidance judgement task forced sentences to be judged either as approach or avoidance, and participants had no neutral options. As mentioned, it could be possible that negation has an effect of weakening approach or avoidance evaluation towards more neutral values. Thus, it would be of interest to use a Likert-like scale to evaluate sentences that enables a graduated approach and avoidance judgement.

In accordance with EST, action understanding either observed or verbally described involves experiential simulation. In short, the comprehender becomes an immersed experiencer of a described situation, and understanding is the vicarious experience of the situation being described from the point of view of the protagonist ([11], see [8, 10]). There is now a certain consensus in that action understanding, either observed or verbally described, is based on a process of simulation that requires a multimodal integration of motor, affective and cognitive components of action experience [38, 8, 10, 39, 40, 11]. In this study, we have proposed that motivation of the protagonist is simulated for action comprehension in the context of sentences, which constitutes a new topic of research from EST. In particular, we have examined the role of negation in approach/avoidance understanding, which opens up new insights into the role of negation in language comprehension from a simulationist point of view. Further research on the role of negation in understanding motivational utterances is necessary. Providing new evidence will lend support to more general theories of simulation during language comprehension, and to the two- step model in particular [2].

## Contributions

Although our study has limitations, it presents some relevant contributions to research into the role of negation in the comprehension of action sentences. On the one hand, it examines the interaction of a category of verbal actions characterized by having a motivational direction with sentential negation. Examination of this interaction seems opportune as approach and avoidance present cognitive communalities with affirmation and negation. In addition, proposals about how approach and avoidance are represented, and how negation affects representation of alternatives in this type of actions seem theoretically relevant and could have an impact on social cognition and communication research. In particular, this could be important in terms of social navigation by representing ourselves and communicating to others our preferences and aversion towards environmental stimuli either in an affirmative or a negative way.

Thus, our results, although exploratory, could help to comprehend the role of negation in understanding motivational and attitudinal actions in social contexts. They support the two-step model of negation [5] and enrich it by examining the effect of implicit negations. This constitutes a different approach to the study of negation through the building up of a representation for sentence comprehension. In this regard, understanding avoidance action sentences has been examined for the first time in accordance with the hypothesis that avoidance verbs are implicitly negative. Within the EST, our study examines affective representation focused on the protagonist’s attitude and motivation in action-sentences understanding. As far as we know, this is a context in which the role of negation has not been studied.

## Conclusions

Motivational direction of utterances refers to approach/avoidance intentionality in our interactions with environmental stimuli expressed by means of verbs (e.g., accept vs reject). Thus, approach and avoidance constitute a semantic frame or category to be encoded to understand motivational sentences, since representing and communicating an individual’s intentional direction towards stimuli has an adaptive and pragmatic role. Likewise, negation is an important linguistic operator. In the context of sentence comprehension, negation either induces inhibition of information under its scope in the situation model or triggers the representation of a positive alternative. We examine the interaction between negation and motivational direction in sentence comprehension in two probe recognition experiments with a 1500 ms and 500 ms delay of recognition probe. Results suggest that at 1500 ms, negation triggers the representation of the positive alternative motivation in approach sentences. By contrast, building up the alternative meaning of negated avoidance sentences seems to imply more complex processing, as avoidance verbs would be implicitly negative. Due to its role as a linguistic operator, negation enriches human capacity for verbally understanding, encoding and communicating representation of individuals’ motivations towards stimuli in everyday actions.

## Supporting information

S1 File Experimental data. (XLSX)
